# Spine surgeon specialty differences in single-level percutaneous kyphoplasty

**DOI:** 10.1186/s12893-019-0630-5

**Published:** 2019-11-06

**Authors:** Kejia Hu, Motao Liu, Amy J. Wang, Gexin Zhao, Yuhao Sun, Chaoqun Yang, Yiwang Zhang, Matthew M. Hutter, Dehong Feng, Bomin Sun, Ziv Williams

**Affiliations:** 1 Center of Functional Neurosurgery, Ruijin Hospital, Shanghai Jiao Tong University School of Medicine, Shanghai, 200025 China; 2 Department of Neurosurgery, Massachusetts General Hospital, Harvard Medical School, Boston, MA 02114 USA; 3 Department of Orthopedics, Wuxi People’s Hospital, Nanjing Medical University, Wuxi, 214023 China; 4 Department of Biomedical Engineering, Columbia University, New York, NY 10027 USA; 5 State Key Laboratory of Oncology in South China, Sun Yat-sen University Cancer Center, Guangzhou, 510060 China; 6 Department of Hand Surgery, Huashan Hospital, Fudan University, Shanghai, 200040 China; 7 Department of Neurosurgery, No. 910th Hospital of The People’s Liberation Army Joint Logistics Support Force, Quanzhou, China; 8 Department of Surgery, Massachusetts General Hospital, Harvard Medical School, Boston, MA 02114 USA

**Keywords:** Percutaneous kyphoplasty, Surgeon specialty, Postoperative complications, Unplanned readmission, Risk predictors, ACS NSQIP

## Abstract

**Background:**

Percutaneous kyphoplasty (PKP) is a procedure performed by a spine surgeon who undergoes either orthopedic or neurosurgical training. The relationship between short-term adverse outcomes and spine specialty is presently unknown. To compare short-term adverse outcomes of single-level PKP when performed by neurosurgeons and orthopedic surgeons in order to develop more concretely preventive strategies for patients under consideration for single-level PKP.

**Methods:**

We evaluated patients who underwent single-level PKP from 2012 to 2014 through the American College of Surgeons National Surgical Quality Improvement Program (ACS NSQIP). We used univariate analysis and multivariate logistic regression to assess the association between spine surgeon specialty and short-term adverse events, including postoperative complication and unplanned readmission, and to identify different independent risk predictors between two specialties.

**Results:**

Of 2248 patients who underwent single-level PKP procedure, 1229 patients (54.7%) had their operations completed by a neurosurgeon. There were no significant differences in the development of the majority of postoperative complications and the occurrence of unplanned readmission between the neurosurgical cohort (NC) and the orthopedic cohort (OC). A difference in the postoperative blood transfusion rate (0.7% NS vs. 1.7% OC, *P* = 0.039) was noted and may due to the differences in comorbidities between patients. Multivariate regression analysis revealed different independent predictors of postoperative adverse events for the two spine specialties.

**Conclusions:**

By comparing a large range of demographic feature, preoperative comorbidities, and intraoperative factors, we find that short-term adverse events in single-level PKP patients does not affect by spine surgeon specialty, except that the OC had higher postoperative blood transfusion rate. In addition, the different perioperative predictors of postoperative complications and unplanned readmissions were identified between the two specialties. These findings can lead to better evidence-based patient counseling and provide valuable information for medical evaluation and potentially devise methods to reduce patients’ risk.

## Background

Percutaneous kyphoplasty (PKP) has become a common surgical technique for the treatment of vertebral compression fractures, including osteoporotic, traumatic, and metastatic compression fractures [1–3]. With the goal of alleviating pain and preventing further loss of vertebral body height or progression of kyphotic deformity, PKP is a modification of percutaneous vertebroplasty (PVP), in which a balloon tamp is inflated in the vertebral body to create a cavity for injection of cement and minimization of extravasation. Although there are several different indications for PKP and PVP, previous studies have suggested that both procedures are effective treatments, and noted an overall trend of increasing utilization of percutaneous vertebral augmentations with a significantly greater use of PKP compared to PVP [4–7].

Despite PKP being a ubiquitous minimally invasive percutaneous technique, adverse events may occur. Understanding risk factors for complications remains a central focus because short-term perioperative complications and hospital readmission is a major burden on the healthcare system [8–10]. Most of the previous studies examining perioperative complications have gravitated towards studying therapeutic approaches, devices, and pharmaceuticals. Using elderly patients (65 years or older) who underwent vertebral augmentation in the the American College of Surgeons National Surgical Quality Improvement Program (ACS NSQIP) database from 2011 to 2012, a study by Toy et al. [11] identified ASA class 4 and inpatient status as risk factors for postoperative complication. History of pulmonary disease and inpatient status were found to be risk factors for unplanned readmission. However, surgeon specialty or practice area as a provider-side factor must also be considered.

Like many other spinal procedures, PKP is often performed by surgeons of two different specialties. When vertebral compression fractures patients are considering having a PKP procedure, one of the most common questions that they or their primary care providers may ask is, “A neurosurgeon or an orthopedic surgeon?” Prior studies have demonstrated that surgeons with training or practice in separate specialties might achieve different outcomes when performing the same operation [12, 13]. The current studies of PKP have all yielded.

mixed data between the two specialties. However, several spine-related studies have argued that past specialty training is not a risk factor for short-term adverse outcomes for spine procedures [14–17]. However, to our knowledge, no report has investigated differences in short-term perioperative outcomes by surgeon specialty for PKP or identified and distinguished risk factors related to outcomes in these patients.

To help address this gap in the reported literature, we utilize the ACS NSQIP database, in which spine surgeon specialty is recorded, for the purpose of discovering whether different adverse 30-day short-term outcome rates in patients following single-level PKP exist between a neurosurgical cohort (NC) and an orthopedic surgery cohort (OC). In addition, we attempt to identify different independent predictors of adverse outcomes for NC and OC, which may enhance evidence-based preoperative strategies and the implementation of accurate risk-reduction decision making.

## Methods

### Data source

The ACS NSQIP database was used and PKP patients from 2012 to 2014 were included. Patients’ data of 121 participating United States hospitals was recorded by the ACS-NSQIP database in 2006 and the number of hospitals has since grown to over 500 by 2014. The data included in the database were identified and collected prospectively by trained professionals from appropriate hospitals of many kind, both academic and private centers, as well as urban and rural, in a prospective systematical and random way. We collected data from patients undergoing major surgical procedures in 30 days after operation, and extracted over 150 patient variables. For example, medical records, operative reports, and patient interviews conducted preoperatively, intraoperatively and postoperatively were include in our analysis [18, 19] We used *Current Procedural Terminology* (CPT) codes 22,523 and 22,524 (Thoracic/Lumbar Percutaneous vertebral augmentation, including cavity creation, fracture reduction and bone biopsy included when performed, using mechanical device, 1 vertebral body, unilateral or bilateral cannulation, inclusive of all imaging guidance) to capture all the patients following single-level spine PKP. Because the data were Health Insurance Portability and Accountability Act (HIPAA)-compliant, this study received an exempt determination from the Massachusetts General Hospital Investigational Review Board.

### Variable selection

We estimated patient-based demographic predictors including hospital status (whether surgery was performed in an inpatient or outpatient setting), age (categorized as ≤64, 65 to 84 and ≥ 85 years old), sex, race, body mass index (BMI, calculated as weight in kilograms divided by the square of height in meters; BMI ≤29.9 kg/m2, not obese; ≥30 kg/m^2^, obese) [20], functional status (independent versus dependent), transfer status (admitted from home vs. admitted from acute care/nursing home/outside emergency department/other), diagnosis, and medical comorbidities (anemia, diabetes, pulmonary disease, cardiovascular disorders, bleeding disorders, blood transfusion≥1 unit packed/whole RBCs within 72 h of operation before surgery, and other system disorders such as disseminated cancer, dialysis use, and steroid use for a chronic condition). We also evaluated surgical risk factors such as the anatomic level anatomic level of operation (thoracic/lumbar region), the American Society of Anesthesiologists (ASA) classification, the type of anesthesia used (spinal/epidural/regional/others versus general) and the operative time.

### Outcomes measure

The primary outcomes we measured were the differences of the development of adverse postoperative events between the two spine specialties cohort, such as systemic complications (occurrence of bleeding transfusion ≥1 unit of packed/whole RBCs given within 72 h post-operatively, deep vein thrombosis, thrombophlebitis, pulmonary embolism, urinary tract infection, pneumonia, unplanned intubation, acute kidney injury, cerebrovascular accident,, sepsis, septic shock, unplanned reoperation and mortality), surgical site wound complications (superficial and deep) within 30-day perioperative duration. We also took unplanned hospital readmission as a second outcome.

### Statistical analysis

The differences in patient and procedure characteristics according to spine surgeon specialty were tested using the χ2, Fisher exact, or Student’s t-test, as appropriate. We used multivariate logistic regression to evaluate risk factors or early predictors and maintained frequencies greater than 10 and also had *P* values < 0.2 in the initial univariate testing [21]. Variables that were missing in more than 20% of the cohort were excluded to avoid model distortion [22]. Odds ratios (OR) and 95% confidence intervals (CI) were reported for both the univariate and multivariate analyses. The variables with *P* values < 0.05 with OR and 95% CI exclusive of 1.0 in the multivariate test were regarded as significant independent predictor [23]. We measured the discriminative power and the goodness of fit of the predictive model using the C-statistic and the Hosmer and Lemeshow test, respectively. All analyses were performed using Stata version 14.0 MP (StataCorp LP, College Station, TX).

## Results

### Participants and descriptive data

A total of 2248 patients who received single level PKP procedures were included and characteristics of the population according to the specialty are provided (Table 1). One thousand two hundred twenty-nine patients (54.7%) were operated on by neurosurgeons. Although there were no significant differences in most of the demographics, preoperative and surgical characteristics, practice patterns differed between NC and OC in several ways. The NC had a larger proportion of Caucasians and African Americans, but a smaller proportion of Asians (*P* < 0.001). Neurosurgeons also performed more PKP operations in emergency cases (*P* = 0.005) and in an inpatient setting (*P* < 0.001). Compared with those in OC, patients in NC were younger (*P* < 0.001), more likely to have independent status (*P* < 0.001), and more likely to have preoperative comorbidities of disseminated cancer (*P* < 0.001) and hypertension requiring medicating (*P* = 0.03).

**Table 1:** Demographics, preoperative, and surgical characteristics of patients undergoing single-level spine percutaneous kyphoplasty

Variable	Orthopedic Surgeon	Neurosurgeon	*P* value †
Available Number (*n* = 2248)	*n* = 1019 (45.3%)	*n* = 1229 (54.7%)	
Demographics			
Hospital status			
Outpatient	653 (64.1)	695 (56.6)	**< 0.001**
Inpatient	366 (35.9)	534 (43.4)	
Age Range, year			
64 or less	128 (12.6)	238 (19.4)	**< 0.001**
65–84	629 (61.7)	721 (58.7)	
85 or greater	262 (25.7)	270 (21.9)	
Sex			
Male	295 (29.0)	369 (30.0)	0.61
Female	724 (71.0)	860 (70.0)	
Body mass index, kg/m^2^ (*n* = 2233)			
BMI ≤ 29.9	780 (77.5)	909 (74.0)	0.071
BMI ≥30	228 (22.5)	318 (25.8)	
Race (*n* = 2132)			
Caucasian	887 (90.4)	1097 (89.3)	**< 0.001**
African American	19 (1.9)	32 (2.6)	
Asian	70 (7.1)	18 (1.5)	
Others	5 (0.5)	4 (0.3)	
Functional status (*n* = 2191)			
Independent	828 (81.2)	1090 (88.7)	**< 0.001**
Dependent	160 (15.7)	113 (9.2)	
Transfer status (*n* = 2245)			
Not transferred (admitted from home)	955 (93.8)	1156 (94.1)	0.689
Transfer from other	63 (6.2)	71 (5.8)	
Diagnosis			
Pathologic fracture	584 (57.3)	618 (50.3)	**< 0.001**
Traumatic fracture	364 (35.7)	517 (42.1)	
Others	111 (10.9)	94 (7.6)	
Preoperative Comorbidities			
Anemia	385 (37.8)	509 (41.4)	0.08
Recent weight loss (> 10% of body weight in last 6 months)	13 (1.3)	19 (1.5)	0.59
All diabetes mellitus			
Insulin-dependent diabetes	74 (7.3)	101 (8.2)	0.441
Non-insulin-dependent diabetes	94 (9.2)	127 (10.3)	
Pulmonary			
Smoking (current smoker within 1 year)	121 (11.9)	157 (12.8)	0.519
Dyspnea	121 (11.9)	163 (13.3)	0.069
Chronic obstructive pulmonary disease	150 (14.7)	176 (14.3)	0.789
Cardiovascular			
Hypertension requiring medicating	692 (67.9)	781 (63.5)	**0.03**
Congestive heart failure	27 (2.6)	27 (2.2)	0.485
Urinary system			
Acute renal failure	12 (1.2)	7 (0.6)	0.117
Dialysis use	3 (0.3)	2 (0.2)	0.509
Other system disease			
Disseminated cancer	29 (2.8)	86 (7)	**< 0.001**
Bleeding disorder	86 (8.4)	88 (7.2)	0.258
Preoperative blood transfusion of ≥1 unit of packed/whole RBCs within 72 h of operation	11 (1.1)	10 (0.8)	0.514
Steroid use for chronic condition	128 (12.6)	150 (12.2)	0.798
Systemic sepsis	26 (2.6)	30 (2.4)	0.867
Operative variables			
Anatomic Level of Operation			
Thoracic Spine	476 (46.7)	611 (49.7)	0.156
Lumbar Spine	543 (53.3)	618 (50.3)	
Wound classification			
Clean	1017 (99.8)	1227 (99.8)	0.851
Clean/contaminated	2 (0.2)	1 (0.1)	
Contaminated	0	0	
Dirty/infected	0	1 (0.1)	
ASA classification (*n* = 2244)			
1-No Disturb	8 (0.8)	7 (0.6)	0.097
2-Mild Disturb	264 (25.9)	287 (23.4)	
3-Severe Disturb	661 (64.9)	796 (64.8)	
4-Life Threat	84 (8.2)	136 (11.1)	
5-Moribund	0	1 (0.1)	
Type of anesthesia (*n* = 2247)			
General	921 (90.4)	1082 (88)	0.085
All others	98 (9.6)	146 (11.9)	
Emergency case	18 (1.8)	46 (3.7)	**0.005**
Operative time, min^a^	33.94 ± 40.99	35.95 ± 34.03	0.2041
Discharge Destination (*n* = 2232)			
Home	823 (80.1)	1016 (82.7)	0.637
Facility Which Not Home	176 (17.2)	206 (16.8)	
Days from Operation to Discharge, day^a^	1.30 ± 2.91	1.61 ± 4.55	0.06

Data are reported as number (percentage) of patients except where noted. For variables with missing data, the number of available observations is given in brackets

*ASA* American Society of Anesthesiologists, *N* number of patients, *SD* standard deviation, *BMI* body mass index

^a^Values are presented as the mean and the standard deviation

†Calculated using ANOVA for continuous variables and chi-square tests for categorical variables, differences were considered significant at *p* < 0.05, and these values are listed in bold type

### Postoperative adverse events

A total of 230 patients (10.2%) had documented one or more postoperative complications while 214 patients (9.5%) had unplanned readmissions within the 30-day postoperative period. NC and OC had similar results for the majority of the postoperative adverse outcomes (Table 2). There was no difference in overall complications (OC 10.6% vs. NC 10.0%, *P* = 0.601), including mortality (OC 2.0% vs. NC 1.5%, *P* = 0.121), systemic complications (OC 6.2% vs. NC 5.9%, *P* = 0.81) or wound complications (OC 0.1% vs. NC 0.2%, *P* = 0.676). The only exception being that OC had a higher occurrence of bleeding requiring transfusions within 72 h postoperatively (OC 1.7% vs. NC 0.7%, *P* = 0.039). Unplanned reoperation rates (OC 3.5% vs. NC 3.7%, *P* = 0.791) were similar, but neurosurgeons performed more reoperation cases that related to the principal PKP procedure (OC 0.4% vs. NC 1.1%, *P* = 0.048) and also had significantly shorter time window between the principal procedure and reoperation (OC 21.54 ± 8.03 day vs. NC 18.0 ± 8.29 day, *P* < 0.001). Unplanned readmission rates (OC 9.2% vs. NC 9.8%, *P* = 0.664) between the two specialties showed no difference. In addition, the NC had shorter times from the principal operation to unplanned readmissions than the OC (OC 14.95 ± 8.55 day vs. NC 13.84 ± 7.60 day, *P* < 0.001).

**Table 2:** Frequency of postoperative complications and outcomes of single-level spine percutaneous kyphoplasty by specialty

Characteristic	Total	OC (*N* = 1019)	NC (*n* = 1229)	*P* value†
Overall Complications	230 (10.2)	108 (10.6)	122 (10.0)	0.601
Mortality in 30d	42 (1.9)	24 (2.0)	18 (1.5)	0.121
Systemic Complications	136 (6.0)	63 (6.2)	73 (5.9)	0.81
Occurrence of bleeding transfusions (≥1 unit of packed/whole RBCs given within 72 h post-operatively)	26 (1.2)	17 (1.7)	9 (0.7)	**0.039**
Pneumonia	25 (1.1)	13 (1.3)	12 (1)	0.5
Pulmonary embolism	15 (0.7)	7 (0.7)	8 (0.7)	0.917
Unplanned intubation	15 (0.7)	7 (0.7)	8 (0.7)	0.917
Ventilator > 48 h	7 (0.3)	3 (0.3)	4 (0.3)	0.895
Stroke/cerebrovascular accident	3 (0.1)	2 (0.2)	1 (0.1)	0.593
Cardiac arrest requiring CPR	5 (0.2)	4 (0.4)	1 (0.1)	0.183
Myocardial Infarction	7 (0.3)	4 (0.4)	3 (0.2)	0.708
Deep venous thrombosis requiring therapy	11 (0.5)	5 (0.5)	6 (0.5)	0.993
Acute renal failure	5 (0.2)	1 (0.1)	4 (0.3)	0.385
Progressive renal insufficiency	5 (0.2)	2 (0.2)	3 (0.2)	0.811
Urinary tract infection	50 (2.2)	19 (1.9)	31 (2.5)	0.292
Sepsis/septic shock	18 (0.8)	8 (0.8)	10 (0.8)	0.94
Wound related Complication	3 (0.1)	1 (0.1)	2 (0.2)	0.676
Unplanned reoperation	82 (3.7)	36 (3.5)	46 (3.7)	0.791
Reoperation related to principal operative procedure	18 (0.8)	4 (0.4)	14 (1.1)	**0.048**
Days from principal operative procedure to Unplanned Reoperation^a^		21.54 ± 8.03	18 ± 8.29	**< 0.001**
Unplanned readmission	214 (9.5)	94 (9.2)	120 (9.8)	0.664
Unplanned Readmission likely related to the principal procedure	74 (3.3)	33 (3.2)	41 (3.3)	0.897
Days from principal operative procedure to Unplanned Readmission^a^		14.95 ± 8.55	13.84 ± 7.60	**0.001**

*CPR* cardiopulmonary resuscitation, *OR* operation room, *N* number of patients, *NC* neurosurgical cohort, *OC* orthopedic cohort

^a^Values are presented as the mean and the standard deviation

†Calculated using ANOVA for continuous variables and chi-square tests for categorical variables, differences were considered significant at *p* < 0.05, and these values are listed in bold type

### Univariate analysis

#### Overall postoperative complication

**Fig. 1:**
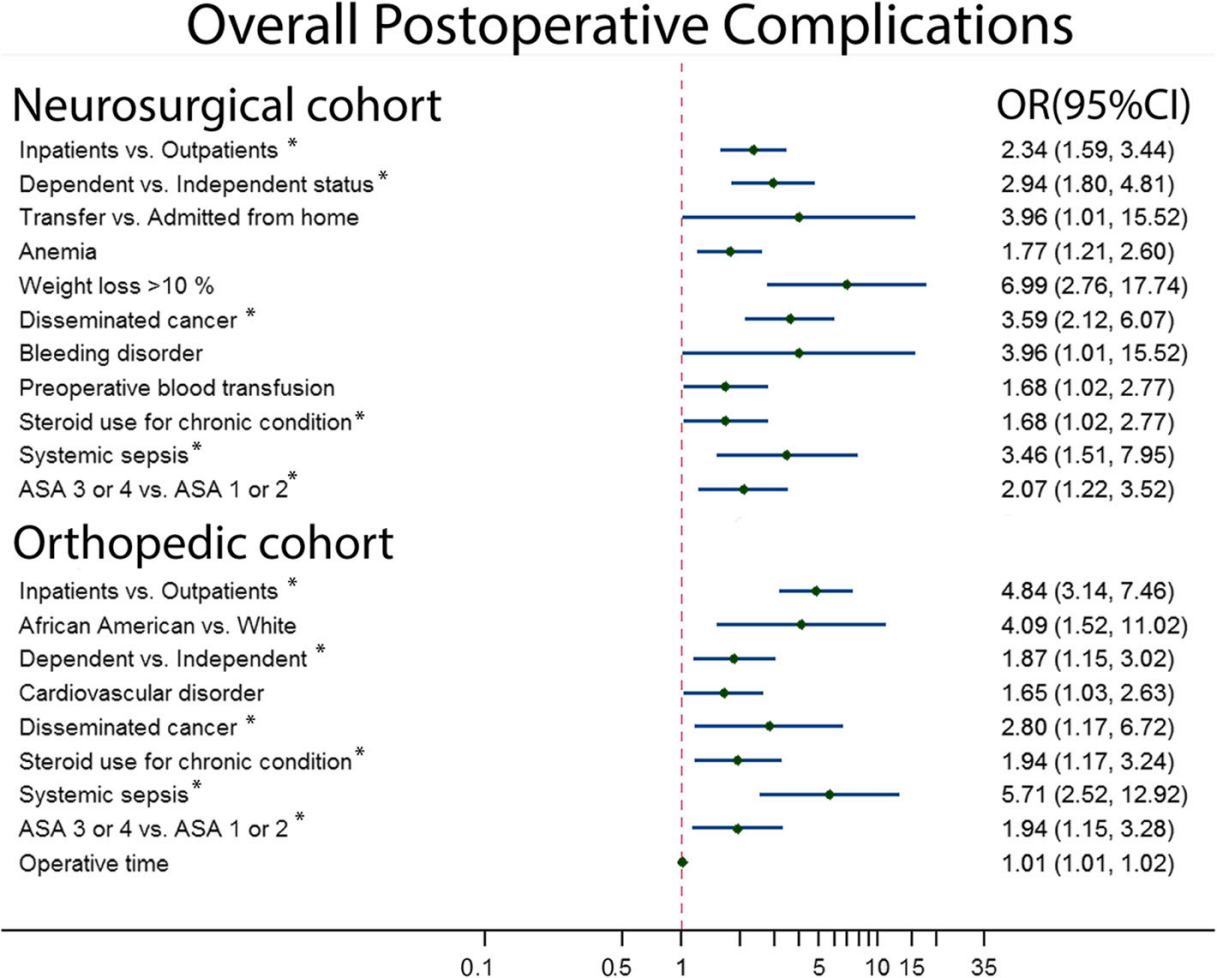
Univariate analysis of factors associated with 30-day overall postoperative complications after single-level percutaneous kyphoplasty between neurosurgical cohort and orthopedic cohort. * presented as same factors between two cohorts

For both specialties, patients with postoperative complications were more likely to be associated with inpatient status for their procedures, have dependent functional status, have disseminated cancer, use steroids for a chronic condition, have systemic sepsis, or be ASA class level 3 or 4. However, transferred status, recent weight loss> 10%, anemia, preoperative anemia, and bleeding disorders were specific for NC. In addition, for OC, development of complications was also associated with black race, cardiovascular disorder, and in those who experienced longer operative durations (Fig. 1).

#### Postoperative blood transfusions

**Fig. 2:**
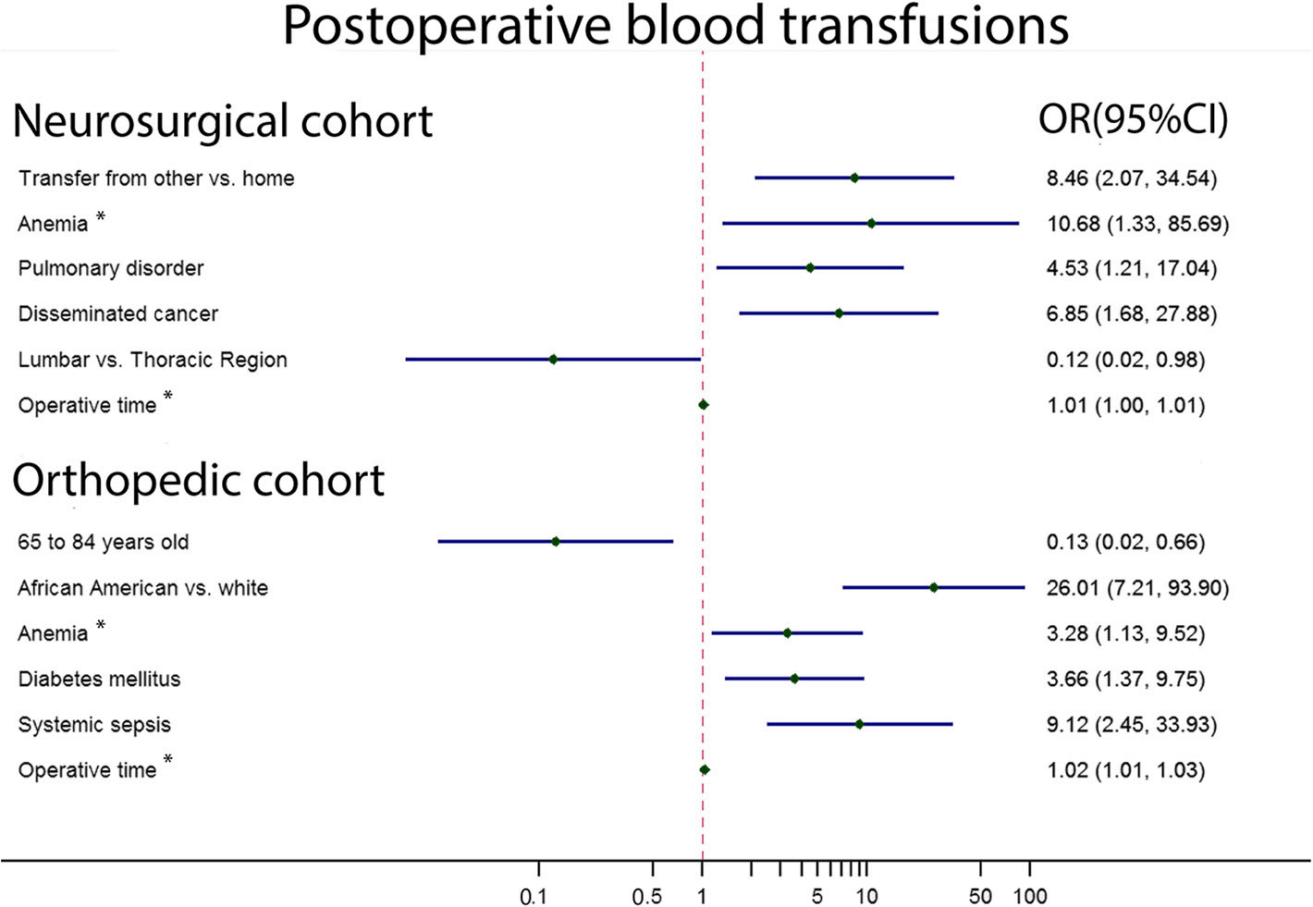
Univariate analysis of factors associated with 30-day Postoperative blood transfusions after single-level percutaneous kyphoplasty between neurosurgical cohort and orthopedic cohort. * presented as same factors between two cohorts

PKP patients with preoperative anemia and longer operative durations had significantly higher chances for requiring postoperative blood transfusions in both specialties. For the NC, transfusions were also associated with having been transferred from another facility not including home, having a preoperative pulmonary disorder, or having disseminated cancer. Interestingly, lumbar PKP had a lower transfusion rate than thoracic PKP. For OC, African Americans had higher transfusion rates compared with Caucasians, in addition those with preoperative diabetes and systemic sepsis. Interestingly, patients aged 65 to 84 were less likely to require a transfusion compared with those aged 64 or less (Fig. 2).

#### Unplanned readmission

**Fig. 3:**
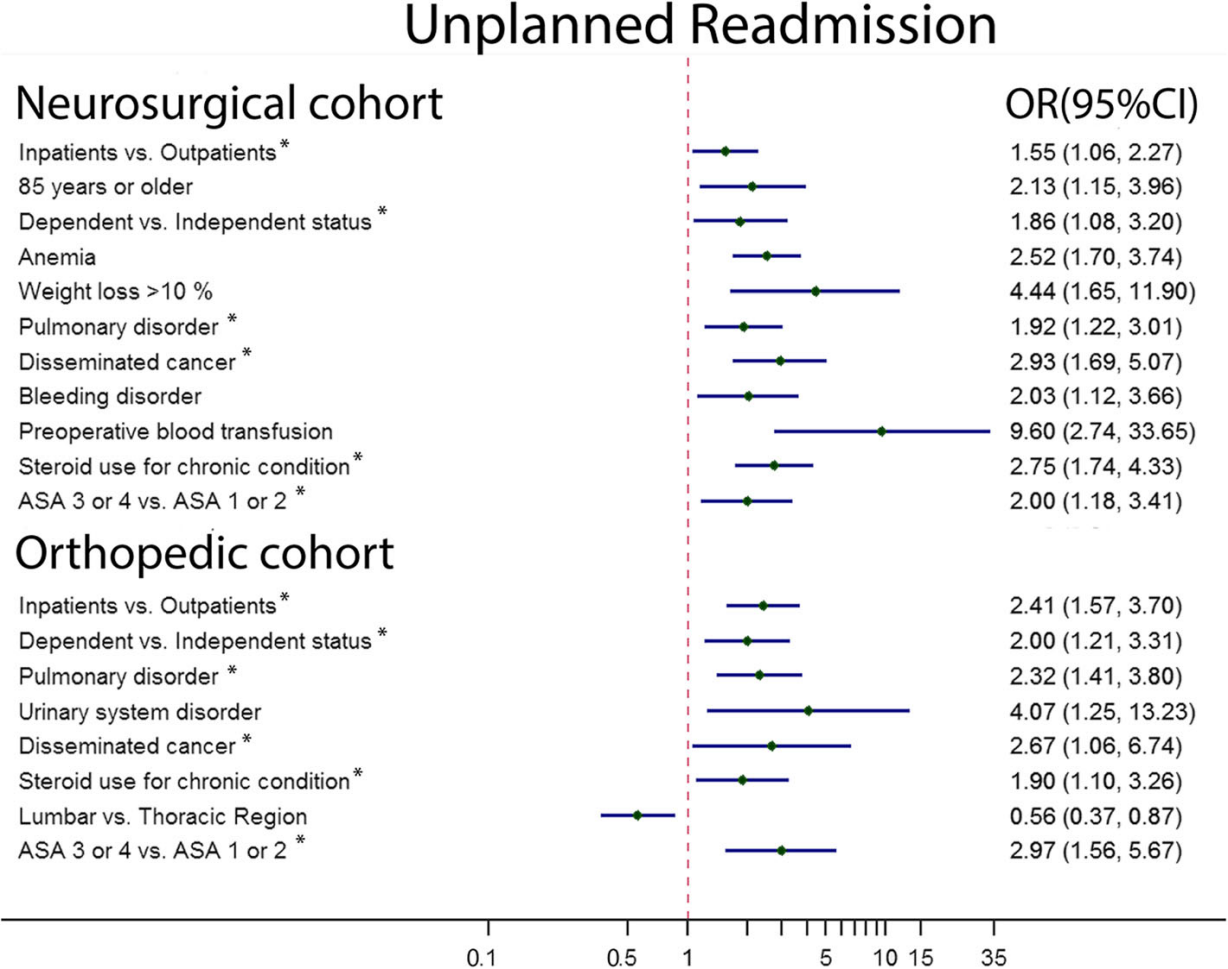
Univariate analysis of factors associated with 30-day unplanned readmission after single-level percutaneous kyphoplasty between neurosurgical cohort and orthopedic cohort. * presented as same factors between two cohorts

Patients with inpatients procedure status, dependent functional status, preoperative pulmonary disorder, disseminated cancer, steroid use for a chronic condition, and ASA class level 3 or 4 all had significantly higher unplanned readmission rates in both specialties. For NC, the additional associated risk factors were being aged 85 or greater, having preoperative anemia, having recent weight loss> 10%, and requiring a preoperative blood transfusion. For OC, having a urinary system disorder also increased the unplanned readmission rates. However, operations in the lumbar region decreased the readmission rates compared with those in the thoracic spine (Fig. 3).

### Multivariate regression models

While controlling for all exposures of interest and despite the cohorts being relatively small conducting a multivariate regression for postoperative transfusion, several factors were independently associated with overall postoperative complication and unplanned readmission rates in OC and NC were identified (Table 3).

**Table 3:** Independent predictors of overall postoperative complications and unplanned readmissions following single-level spine percutaneous kyphoplasty treated by neurosurgeons and orthopedic surgeons

Surgeon Specialty	Independent Predictors	Odds ratio	95% Confidence Interval		*P* value*	C-statistic
Overall Postoperative Complication						
NC	Inpatients vs. Outpatients	1.874	1.216	2.889	0.004	0.7005
	Dependent vs. Independent	2.418	1.424	4.107	0.001	
	Disseminated cancer	2.743	1.511	4.979	0.001	
	Weight loss > 10% in 6 months	3.867	1.387	10.780	0.01	
OC	Inpatients vs. Outpatients	3.187	1.968	5.159	< 0.001	0.7559
	Systemic sepsis	3.688	1.365	9.964	0.01	
	Operative duration	1.010	1.004	1.016	0.001	
Unplanned Readmission						
NC	Anemia	1.993	1.298	3.058	0.002	0.7109
	Disseminated cancer	2.160	1.168	3.994	0.014	
	Preoperative blood transfusion	7.919	1.922	32.632	0.004	
	Steroid use for chronic disorder	2.203	1.352	3.589	0.002	
OC	Inpatients vs. Outpatients	2.187	1.356	3.527	0.001	0.7175
	Urinary system disorder	3.728	1.061	13.094	0.04	
	Lumbar vs. Thoracic Region	0.594	0.370	0.953	0.031	
	ASA 3 or 4 vs. ASA 1 or 2	2.139	1.020	4.486	0.044	

*NC* neurosurgical cohort, *OC* orthopedic cohort, *ASA* American Society of Anesthesiologists class

*Significance, *P* ≤ 0.05

#### Neurosurgeon PKP cases

For the NC, PKP patients who had inpatient status (OR = 1.87, *P* = 0.004), dependent functional status (OR = 2.42, *P* = 0.001), had preoperative disseminated cancer (OR = 2.74, *P* = 0.001), or who had recent weight loss (> 10% of body weight in last 6 months) (OR = 3.87, *P* < 0.001) were at increased odds of postoperative complications compared with controls. Preoperative anemia (OR = 1.99, *P* = 0.002), disseminated cancer (OR = 2.16, *P* = 0.014), steroid use for chronic disorder (OR = 2.20, *P* = 0.002) and blood transfusion (OR = 19.69, *P* < 0.001) showed a strong trend toward predicting unplanned readmission.

#### Orthopedic surgeon PKP cases

For the OC, longer operative durations (OR = 1.01, *P* = 0.001), preoperative systemic sepsis (OR = 3.69, *P* = 0.01), and inpatient status (OR = 1.87, *P* = 0.004) in PKP patients were all significantly associated with overall postoperative complication. The independent predictors of unplanned readmissions were inpatient status (OR = 2.19, *P* = 0.001), preoperative kidney disorder (OR = 3.73, *P* = 0.04), and ASA 3 or 4 (OR = 2.14, *P* = 0.044), but operations in the lumbar region (OR = 0.59, *P* = 0.03) showed a decreased odds of unplanned readmission comparing with those in the thoracic spine.

The C-statistics for the final regression models (0.7005, 0.7559, 0.7109 and 0.7175, respectively) indicated good discriminative capacities, and the goodness-of-fit tests showed no statistically significant lack of fit, demonstrating good calibrations between all the models and the source data.

## Discussion

In the current health care environment, quality outcomes are critical metrics of patients’ satisfaction. Identification of risk predictors of short-term adverse events is important for risk stratification and tailored therapy for patients. Toy et al. [11] reported an overall postoperative complication rate of 9.5% in 850 patients, with death occuring in 1.5% of patients and 10.8% of patients requiring readmission. Our study, while being specific to single-level PKP and being derived from a different period data set from ACS NSQIP database, demonstrated similar results, Our study was also able to distinguish several significant differences in the baseline clinical demographics of PKP patients between the OC and the NC. Considering these baseline differences, it would be natural to hypothesize that risk factors for adverse events would also be different. However, to date, there has been a lack of studies primarily focusing on the difference of risk predictors among spine patients according to surgeon specialty. To the best of our knowledge, this is the first report identifying different risk predictors for short-term adverse events following PKP by spine specialty. Our study confirmed that postoperative complications and unplanned readmissions between the two specialties most often performing single-level PKP were almost indistinguishable, which suggests that spine surgeon specialty may not affect the majority of short-term adverse events in PKP patients. While there is no significant difference in mortality between the NC and the OC according to previous studies, 30-day perioperative mortality has been reported as low as 0.13% in kyphoplasty [24] and 0.57% in vertebroplasty [25] for osteoporotic vertebral fractures, both of which are dramatically lower than in our study. This high mortality rate following PKP may be due to the older population comprising majority of the surgical cohort (83.7%) and the high rates (approximately 50%) of preoperative co-morbidities in this population. Our study suggests that the 30-day mortality (1.9%) following PKP was similar to other spine surgical procedures, when comparing with the NSQIP dataset source [17] and other dataset sources (including those from USA (1.4%) [26] or Europe (2.7%) [27]), despite the minimally invasive nature of PKP.

The only difference in postoperative complications between the OC and the NC is the occurrence of bleeding requiring transfusion. Several past articles comparing neurosurgeon and orthopedic surgeons in spine surgery have also reported this postoperative transfusion difference [14–17]. PKP was developed as a minimally invasive procedure to avoid blood loss compared with open surgery; it is interesting that similar to other spine procedures, the difference persists between the two specialties. For specific hematopoietic system disorders, there were no significant differences in preoperative anemia, bleeding disorders and those who received preoperative blood transfusion between the OC and the NC. Using univariate analysis, we found that only preoperative anemia was associated with postoperative transfusion for both specialties. Although there are an insufficient number of cases to do a multivariate regression analysis to find independent predictors of transfusion requirements for each specialty, several reasons may be cited for the discordance in postoperative transfusion rates between the OC and the NC. First, it may be reflective of the comorbidity burden in these patients and not the specialty itself. We identified some differences in comorbidities between patients being treated by these two specialties, but it remains hard to quantify which cohorts overall condition is worse. For example, the NC had a higher proportion of inpatients, patients with disseminated cancer, and emergency cases, while the OC had higher rates of patients with dependent status and hypertension requiring medication. A second explanation may be the differences in the frequency of underlying spine pathologies. For instance, the OC had a higher percentage of pathologic fractures while the NC had higher rates of disseminated cancer. However, pathologic vertebral fractures due to degenerative disease such as osteoporosis may also have different bleeding tendencies compared with tumor-related metastatic malignant lesions, traumatic fractures have widely varying bleeding rates depending on the extent of bony injury, which increase the complexity of analysis. Given the limitations of the NSQIP database, it is difficult to determine the relationship between these two. A final reason it may be difficult to elucidate the relationship between post-operative transfusions in these two cohorts is that each specialty may have different thresholds and criteria for which a blood transfusion occurs. At present there are no clear universal transfusion protocols and the degree to which postoperative anemia can be tolerated may differ for both specialties. In addion, unnecessary transfusions may increase other complications and contribute to wasting limited medical resources [28], making comparisons of blood-preservation strategies between the two specialties challenging.

With multivariate regression analyses, our study showed the noteworthy finding that between the two specialties, almost all the independent risk factors associated with overall postoperative complication and unplanned readmission after PKP are different. Inpatient status is the only risk factor for postoperative complications shared by both specialties, which is concordant with a previous vertebral augmentation study [11], and may help further signify its role in risk stratification. In addition, anemia and obtaining a preoperative blood transfusion were independent risk factors for unplanned readmission in the NC but not the OC. The variations in independent predictors for postoperative adverse events between the OC and the NC may reflect specialty differences in patient management and may have implications for perioperative management of PKP patients. Understanding these risk factors may not only enhance spine surgeons’ knowledge but also lead to better evidence-based patient counseling regarding expected adverse events during admission and what may occur after discharge. In addition, simple yet effective measures including patient education regarding the risks and types of adverse events to expect, rigorous early in-person or telephone follow-up, monitoring of surgical sites, and coordinated post-discharge care via mid-level providers may reduce the incidence of these complications and readmission rates, subsequently leading to decreased healthcare expenditures [29].

Similar to other NSQIP database studies, several limitations of our study deserve mention. First, the postoperative complications tracked by NSQIP database are not specific to PKP procedure or spine surgery but are instead generalized across all surgical specialties. Moreover, several PKP procedure-specific adverse events are not available in the database, because they are either too specific or not evident at 30 days, such as cement leakage or extravasation, cement embolisms, allergic reactions to cement, hematoma, vertebral fractures at adjacent levels and neurological compromise. These cannot be clarified with the NSQIP database, which may skew the data towards a higher postoperative complication incidence. Furthermore, in many parts of the world interventional radiologists also perform PKP procedures, which were not compared here. Second, the NSQIP dataset does not have exact and precise information on the standardized comorbidity scores. Here, we attempted to control for the presence of comorbid disease by using the ASA class and the total number of different system comorbidities as covariates. Third, there are likely factors that were not investigated in NSQIP database, such as insurance status and other socioeconomic factors which may also be factors that influence readmission [30]. Fourth, the presence of missing data has the potential to influence conclusions substantially, so we employed similar practices that others have used to handle NSQIP dataset including the complete case analysis, which involves analysis of only those patients for whom data exist for all predictors of interest and all other cases are excluded from evaluation. However, the importance of using missing values carefully still needs to be highlighted [22]. Finally, although the final model was well calibrated to the data, the number of patients who had postoperative adverse events was comparatively small; therefore, it is difficult to build a stable multivariate regression model for the risk factors in the subgroup of each specific complication, so prospective studies with PKP metrics are needed to fully understand the scope of the postoperative adverse events. Nonetheless, the ACS NSQIP database still provides some important advantages that shed light on the short-term perioperative setting and its specific relationship to single-level PKP procedures.

## Conclusion

Using a nationwide cohort across a large range of demographic features, preoperative comorbidities, and intraoperative factors, our findings suggest that spine surgeon specialty does not affect short-term adverse events in single-level PKP patients, except that the OC had higher postoperative blood transfusion rates. In addition, the different perioperative predictors of postoperative complications and unplanned readmissions were identified between the two specialties. These findings can help improve our understanding of these potential perioperative issues that can arise in single-level PKP patient population treated by different spine surgeons. With early recognition, this information can assist spine surgeons and related healthcare providers in determining which patients may merit further preoperative medical evaluation while striving for minimizing the chances of postoperative adverse events and triaging PKP patients at discharge for optimal follow-up.

## Data Availability

The data that support the findings of this study are available from ACS-NSQIP but restrictions apply to the availability of these data, which were used under license for the current study, and so are not publicly available. Data are however available from the authors upon reasonable request and with permission of ACS-NSQIP.

## Abbreviations

ASA: American Society of Anesthesiologists
BMI: Body mass index
NC: Neurosurgery cohort
OC: Orthopedic cohort
PKP: Percutaneous kyphoplasty
SD: Standard deviation
